# Home-Based HIIT and Traditional MICT Prescriptions Improve Cardiorespiratory Fitness to a Similar Extent Within an Exercise Referral Scheme for At-Risk Individuals

**DOI:** 10.3389/fphys.2021.750283

**Published:** 2021-11-10

**Authors:** Katie Hesketh, Helen Jones, Florence Kinnafick, Sam O. Shepherd, Anton J. M. Wagenmakers, Juliette A. Strauss, Matthew Cocks

**Affiliations:** ^1^Research Institute for Sport and Exercise Science, Liverpool John Moores University, Liverpool, United Kingdom; ^2^School of Sport, Exercise and Health Sciences, National Centre for Sport and Exercise Medicine, Loughborough University, Loughborough, United Kingdom

**Keywords:** high-intensity interval training, cardiorespiratory fitness, heart rate, exercise adherence, exercise referral scheme, primary care, body composition

## Abstract

Exercise referral schemes (ERS) are used to promote physical activity within primary care. Traditionally, ERS are conducted in a gym or leisure-center setting, with exercise prescriptions based on moderate-intensity continuous training (MICT). Home-based high-intensity interval training (Home-HIIT) has the potential to reduce perceived barriers to exercise, including lack of time and access to facilities, compared to traditional MICT prescription used with ERS and improve health related outcomes. We hypothesized that Home-HIIT would mediate greater improvement in cardiorespiratory fitness (CRF) by virtue of greater adherence and compliance to the exercise prescription, compared to MICT.

**Methods:** Patients enrolled on an ERS (Liverpool, United Kingdom) were recruited for a pragmatic trial. Participants self-selected either 12 weeks of MICT (45–135 min/week at 50–70% HR_max_) or Home-HIIT (4–9 min × 1 min intervals at ≥80% of HR_max_, interspersed with 1 min rest). The primary outcome was the change in CRF (VO_2__peak_) at post-intervention (12 weeks) and follow-up (3-month post intervention), using intention-to-treat analysis.

**Results:** 154 participants (age 48 ± 10y; BMI 30.5 ± 6.1 kg/m^2^) were recruited between October 2017 and March 2019, 87 (56%) participants chose Home-HIIT and 67 (44%) MICT. VO_2__peak_ increased post-intervention in both groups (MICT 3.9 ± 6.0 ml.kg^–1^.min^–1^, Home-HIIT 2.8 ± 4.5 ml.kg^–1^.min^–1^, *P* < 0.001), and was maintained at follow-up (*P* < 0.001). Fat mass was only reduced post MICT (MICT −1.5 ± 6.3 kg, *P* < 0.05, Home-HIIT −0.2 ± 2.0 kg, *P* = 1.00), but the reduction was not maintained at follow-up (MICT −0.6 ± 5.1 kg, Home-HIIT 0.0 ± 2.2 kg, *P* > 0.05). Adherence to the prescribed programs was similar (MICT 48 ± 35%, Home-HIIT 39 ± 36%, *P* = 0.77).

**Conclusion:** This is the first study to evaluate the use of Home-HIIT for individuals in a primary care setting. Contrary to our hypothesis, adherence to both exercise prescriptions was poor, and CRF improved to a similar extent in both groups with improvements maintained at 3-month follow-up. We provide evidence that, although not superior, Home-HIIT could be an effective and popular additional exercise choice for patients within primary care based ERS.

**Clinical Trial Registration:** [ClinicalTrials.gov], identifier [NCT04553614].

## Introduction

The World Health Organization (World Health Organization, 2013) and national governments have prioritized the promotion of regular physical activity (PA) as part of a coordinated approach to reduce non-communicable diseases. Primary care is a key setting for the promotion of PA, with exercise referral/physical activity on prescription schemes (ERS) an approach being implemented in various countries (Arsenijevic and Groot, 2017). There are some promising ERS examples [e.g., Wales National ERS (Murphy et al., 2012)] but there is little evidence supporting the efficacy of ERS to improve markers of health (Prior et al., 2019). More precisely, it appears important to consider cardiorespiratory fitness (CRF), a strong predictor of all-cause mortality (Lee et al., 2010). Moreover, uptake and adherence to the exercise programs prescribed by ERS is generally poor (Morgan, 2005; Pavey et al., 2012).

Traditionally, an ERS is carried out in a gym or leisure-center setting and exercise prescriptions are based on traditional exercise guidelines using moderate-intensity continuous training (MICT) (Rowley, 2019). Previous studies have reported barriers to traditional exercise guidelines such as; lack of time, tedious nature of the exercise prescription, lack of access to facilities and poor weather (Hoare et al., 2017). Additionally, barriers specific to the gym environment have been identified including; shame of exercising in front of others, expensive cost of memberships and lack of transport (Chinn et al., 1999). Many of these barriers have also been reported within ERS (Morgan et al., 2016), contributing to poor uptake and adherence (Pavey et al., 2012; Morgan et al., 2016).

Low-volume high-intensity interval training (HIIT) has been shown to elicit improvements in CRF comparable to MICT, despite a substantially lower time commitment (Gibala et al., 2012). In addition, two recent studies have shown HIIT can improve CRF (Jung et al., 2020) and body composition (Roy et al., 2018) in real-world settings, where exercise was completed outside the laboratory without supervision. Therefore, HIIT may address “lack of time,” one of the most commonly cited barriers to PA (Morgan et al., 2016). Recently, HIIT has been successfully modified using simple body-weight exercises, to allow training to be completed at home without equipment (Scott et al., 2019b). This home-based HIIT (Home-HIIT) approach has been shown to reduce exercise barriers (Scott et al., 2019a) and have high adherence when piloted in sedentary obese individuals (Scott et al., 2019b). However, whether HIIT can form a viable public health strategy for use within primary care has been questioned by public health researchers, who cite the strenuous nature of the exercise, and complex protocols as additional barriers (Biddle and Batterham, 2015).

To date, no study has evaluated Home-HIIT as an exercise prescription for ERS, comparing changes in CRF, adherence and compliance to a traditional ERS, where MICT is prescribed. We hypothesized that Home-HIIT would mediate greater improvement in CRF by virtue of greater adherence and compliance, compared to MICT prescription. A multidisciplinary approach was used to explore participant experiences of MICT and Home-HIIT within an ERS.

## Materials and Methods

### Study Design and Participants

The study used a pragmatic design where patients were recruited from the Active Lifestyle ERS in the Metropolitan Borough of Sefton, funded by Sefton Public Health Team, and Liverpool John Moores University ERS. Participants were self-allocated to one of two groups: MICT or Home-HIIT (further details of the self-allocation process, and the rationale for this, can be found in the procedures section of the methods). Eligible participants (Supplementary Data Table 1) provided written informed consent, and the study was approved by the Liverpool Central NHS Research Ethics Committee (17/NW/0042) and conformed to the Declaration of Helsinki.

### Procedures

The study was embedded into an existing ERS which followed United Kingdom National Institute for Clinical Excellence (NICE) guidance for ERS (National Institute for Clinical Excellence, 2014). Patients were invited to 3 meetings with their Lifestyle Development Officer (LDO), (1) initial meeting (week 0), where barriers and motivation to exercise were explored and an exercise prescription was developed, (2) mid-intervention (6 weeks), where progress/barriers toward the exercise prescription was explored, and (3) post-intervention (12 weeks), where strategies to maintain physical activity were discussed. The support provided to the groups was identical, only the exercise prescription differed.

**FIGURE 1:**
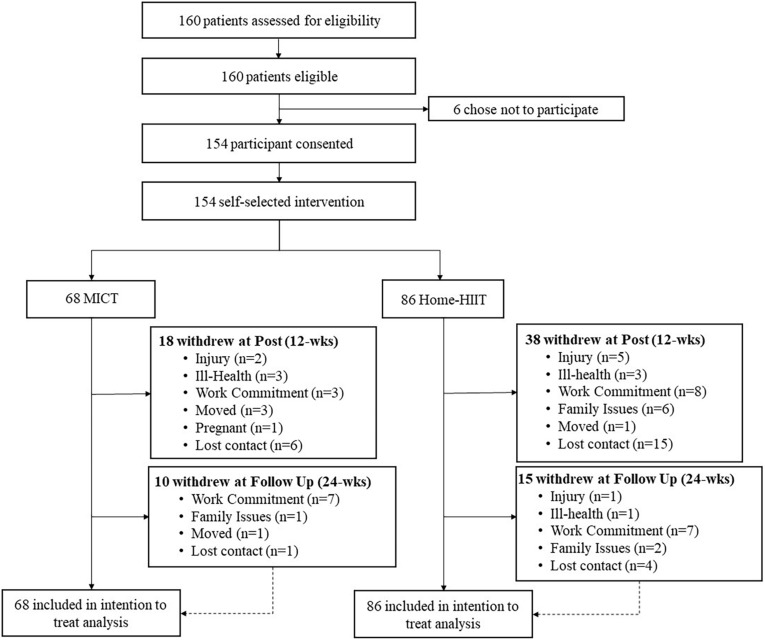
Trial profile diagram.

During the initial, meeting patients were given the choice between a structured exercise prescription or general PA advice. Those who opted for structured exercise were invited to participate in the study. Participants who consented explored their barriers and motivation to exercise. Once this review had been completed, participants were given information on the two exercise prescriptions (MICT or Home-HIT). Participants were then allowed to choose their exercise prescription, based on which fitted their current lifestyle (Figure 1). The information provided to participants was identical and conducted by the same member of the research team. The study hypothesis (i.e., greater CRF gains and adherence in Home HIIT) was not part of the explanation. To minimize potential allocation bias, recruitment to the groups was not restricted at any point; i.e., groups were left open for recruitment until appropriate participant numbers were achieved in both groups. Self-selection of the exercise prescription was chosen to increase the real-world translation of the findings, as patient choice would dictate exercise prescription within existing ERS.

Following the initial meeting, participants attended the laboratory for a baseline assessment after an overnight fast, having abstained from caffeine for 4 h and alcohol and vigorous exercise for 24 h. The same assessment was conducted following the 3-month ERS (post-intervention) and 3-months after the end of the ERS (follow-up), 6-month total study duration. Following 20 min rest, blood pressure (Dinamap; GE Pro 300V2, Tampa, Florida) was measured in triplicate (Scott et al., 2019b). Venous blood samples were taken to determine fasting glucose and blood lipids before an oral glucose tolerance test (OGTT) was performed (Scott et al., 2019b) with samples taken at 60- and 120 min. Body composition was assessed using Dual-energy X-ray Absorptiometry (DXA Hologic QDR Series, Discovery A, Bedford, MA, United States) (Scott et al., 2019b). Finally, an incremental exercise test to exhaustion on a cycle ergometer was administrated to determine CRF (VO_2__peak_). Briefly, participants started cycling at 25W for females or 65W for males for 3 min, following this the workload was increased by 35 W every 3 min until volitional fatigue (Scott et al., 2019b). VO_2__peak_ was determined using an online gas collection system (Moxus modular oxygen uptake system, AEI technologies, Pittsburgh, PA, United States). VO_2__peak_ was defined as the highest VO_2_ achieved over a 15 s recording period. The same experimenter conducted all experimental trials and was not blinded to the group allocation.

Following the post-intervention meeting participants completed an anonymous online qualitative survey^1^ exploring barriers and facilitators to exercise before the ERS, experiences of the exercise intervention (e.g., facilitators and barriers to adherence), and intentions to exercise in the future (Supplementary Data Table 2.). Open ended questions were developed, piloted within, and revised by the research team using appropriate literature (Scott et al., 2019a).

### Traditional Exercise Referral Scheme

Participants were encouraged to train at least 3 x/week, totaling 45 min/week during weeks 1–2 and 135 min/week by week 12 (Table 1). Depending on the participant’s preference, the exercise prescription included use of gym equipment, exercise classes or exercise in the local environment (e.g., walking groups). All prescriptions were based on MICT and following a 5 min warm-up participants were advised to exercise at 50–70% of predicted heart rate maximum (HR_max_; 220–age). Those wanting to use gym equipment, attended their local gym for an induction.

**TABLE 1:** Prescribed exercise for home based high-intensity interval training (Home-HIIT) and moderate-intensity continuous exercise (MICT).

Week	MICT				Home-HIT				
	Total time per session (min)	Intensity (%HR_max_)	Sessions per week	Weekly time commitment (min)	Total time per session (min)	Number of intervals per session	Interval intensity (%HR_max_)	Sessions per week	Weekly time commitment (min)
1	15	50–70	3	45	8	4	≥80	3	24
2	15			45	8	4			24
3	15			45	10	5			30
4	15			45	10	5			30
5	15			45	12	6			36
6	30			90	12	6			36
7	30			90	14	7			42
8	30			90	14	7			42
9	30			90	16	8			48
10	30			90	16	8			48
11	30			90	18	9			54
12	45			135	18	9			54

*Total time per session and total time per week not including warm up and cool down. %HR_max_ percentage of predicted heart rate maximum, when heart rate maximum is calculated using 220-age.*

### Home-High-Intensity Interval Training

Participants were encouraged to train 3x/week. Following a 2 min warm-up of jogging on the spot sessions included repeated 1 min bouts of exercise interspersed with 1 min rest. An interval comprised two different bodyweight exercises for 30 s each, with no rest in between. Participants completed 4 intervals during weeks 1–4, increasing by 1 interval every fortnight up to 9 intervals. Participants were free to choose the specific body-weight exercises from a list of 18 individual exercises (Table 2). During the intervals participants were advised to achieve ≥80% of HR_max_.

**TABLE 2:** List of home-HIIT exercises.

Number	Exercise pairs
1	Mountain Climbers
2	Floor Jacks
3	Get Ups
4	Lateral Jumps
5	Elbow to Knee
6	Jogging Boxers
7	Jogging on the Spot
8	Squat Jumps
9	X-Jumps
10	Jumping Jacks
11	Clapping Jacks
12	Squat Thrusts
13	Split Squats
14	Burpees
15	Jogging with High Knees
16	Spotty Dogs
17	Jump Overs
18	Tuck Jumps

### Follow-Up Period

During the 3-month follow-up period no structured exercise plan was provided, but participants did discuss maintenance of physical activity levels in the final meeting with their LDO.

### Training Session Monitoring

All participants were given a HR monitor (Polar H10) that provided real-time feedback on HR during sessions using an accompanying App^2^. Provision of HR monitors is not normal practice within the Active Lifestyle ERS but HR was essential for providing information on adherence and compliance to the prescriptions. Following training, HR data was automatically uploaded to a cloud storage site^3^ accessible to the participant and research team throughout the intervention.

Using the data on www.flow.polar.com exercise duration and mean HR were recorded for each MICT session. Number of intervals completed, peak HR on each interval, % of intervals achieving the criterion HR (≥80% HR_max_), and time spent above the criterion HR were recorded for each Home-HIIT session. All HR variables were expressed as % of predicted HR_max_.

Using the data on www.flow.polar.com the following metrics of adherence and compliance were assessed:

#### Training Drop-Off

Defined as the week during which participants no longer completed any training sessions. Participants who did not complete any training sessions were described as no exercise uptake.

#### Weekly Adherence

Used 3 sessions as the maximum that could be executed during a week (i.e., 3 or 4 sessions would both = 100% adherence), the mean weekly adherence (%) was then calculated. Weekly adherence was used to account for drop-off and participants who completed more than the prescribed number of sessions in some weeks.

#### Compliance

Is defined differently for MICT and Home-HIIT, but generally refers to the achievement of both a prescribed duration and intensity. For MICT, duration was adjusted for the exercise intensity to produce a HR physical activity score (HRPAS = min^∗^%HR_max_) for each session (Miller et al., 2014). If the session HRPAS was equal to or greater than the prescribed-HRPAS, the session was compliant. Home-HIIT compliance was defined as achieving a HR ≥ 80% HR_max_ during the session and performing the prescribed number of intervals (Table 1).

### Outcomes

The primary outcome was change in CRF following the 12 week intervention, in the MICT group compared with the Home-HIIT group. The secondary outcomes were body composition, blood pressure, glucose tolerance, blood lipids, adherence and compliance to the program and perceptions of the intervention.

### Statistical Analysis

The primary outcome was CRF and the primary comparison was change in CRF between 12 weeks of MICT compared to Home-HIIT. The *a priori* power analysis indicated that 64 participants per group would be required to detect a 1.5 ml/kg/min difference in CRF with a power of 80% to detect a between group difference, assuming a standard deviation for the change in CRF of 3 ml/kg/min (Scott et al., 2019b).

All physiological outcomes were analyzed using intention-to-treat principles, where all participants who consented, regardless of adherence, compliance, or attendance at testing sessions were included. A linear mixed model was used to assess the change in outcomes at post-intervention and follow-up (relative to baseline which was included in the statistical model as a covariate) within and between each treatment group. Time was considered as a categorical variable and an unstructured covariance matrix, which was allowed to differ by treatment group, was used to model the correlation over time.

Descriptive training session data, adherence and compliance were assessed using an independent samples *T*-test. Both intention-to-treat and per-protocol principles were used, as recommended for evaluation of exercise training intervention fidelity (Taylor et al., 2015). For intention-to-treat analysis, all consented participants were included, and it was assumed that missing HR data represented a missed training session. As such, a value of 40% HR_max_ was used to represent no additional physiological load, as previously proposed (Taylor et al., 2015). When reporting per-protocol data for weekly adherence, those defined as no exercise uptake (i.e., had an adherence of 0%) were excluded, but all other consented participants were included. For compliance per-protocol analysis, only data for completed training sessions are presented. Statistical analysis was performed using SPSS and statistical significance set at *P* ≤ 0.05. Data are presented as mean ± SD, unless otherwise stated. The qualitative survey responses were analyzed using a framework approach (Ritchie and Spencer, 2002) which has been used previously within mixed method studies (Scott et al., 2019a). This trial was registered with ClinicalTrials.gov, number NCT04553614.

## Results

### Participant Characteristics

Between Oct 1, 2017 and March 31, 2019, 160 eligible patients were screened, of whom 154 participants were recruited, and of these 68 chose MICT and 86 Home-HIIT (Figure 1). Baseline characteristics are reported in Table 3. At baseline, CRF was significantly lower in Home-HIIT compared to MICT (*P* = 0.038). All other baseline characteristics between groups were similar.

**TABLE 3:** Participant characteristics.

Variable	Intervention		
	All (*n* = 154)	MICT (*n* = 67)	Home-HIIT (*n* = 87)
Age (years)	48 ± 10	48 ± 11	49 ± 10
Sex (male/female)	88/66	41/27	47/39
Height (cm)	171.0 ± 8.7	170.9 ± 8.8	171.3 ± 8.6
Weight (kg)	89.1 ± 17.7	89.5 ± 18.3	88.8 ± 17.4
BMI (kg.m^2^)	30.5 ± 6.1	30.7 ± 6.3	30.3 ± 6.0
VO_2__peak_ (l.min^–1^)	2.2 ± 0.7	2.3 ± 0.7	2.2 ± 0.7
VO_2__peak_ (ml. min^–1^.kg^–1^)	25.6 ± 7.6	26.7 ± 8.4	24.6 ± 6.9*
Watt Max (W)	159 ± 52	164 ± 51	155 ± 52
**Health Condition (*n*=)**			
Anxiety	3	2	1
Arthritis	12	7	5
Asthma	13	5	8
Bronchiectasis	1	0	1
Chronic Kidney Disease	3	0	3
Depression	8	3	5
Dyslipidemia	74	31	43
Fibromyalgia	1	0	1
Hypertension	34	17	27
Impaired Fasting Glucose	5	3	2
Impaired Glucose Tolerance	16	10	6
Kidney Stones	1	1	0
Obesity	69	27	42
Polycystic Ovary Syndrome	2	0	2
Sedentary	139	59	80
Sleep Apnea	2	2	0
Thyroid Condition	5	0	5
Mean number of risk factors per participant	3 ± 1	3 ± 1	3 ± 1
Range of Risk Factors	1–7	1–6	1–7

*Indicates a significant value.

### Cardiorespiratory Fitness

Cardiorespiratory fitness (absolute and relative VO_2__peak_) significantly increased post-intervention (MICT *P* < 0.001, Home-HIIT *P* < 0.001) and at follow-up (MICT *P* = 0.007, Home-HIIT *P* = 0.047; Table 4). There were no significant between-group differences at either time point (Post-intervention *P* = 0.130, Follow-Up *P* = 0.208). Wattmax significantly increased post-intervention (MICT *P* < 0.001, Home-HIIT *P* = 0.035) and at follow-up (MICT *P* < *0.001*, Home-HIIT *P* = 0.023; Table 4), with no significant between-group differences at either time point (Post-intervention *P* = 0.108, Follow-Up *P* = 0.178).

**TABLE 4:** Changes to cardiorespiratory fitness, body composition cardiovascular responses, glucose tolerance and blood lipid responses post-intervention and at 3-month follow-up compared to baseline.

Variable	Time	MICT	*P Value*	Home-HIIT	*P Value*	Between group differences (95%CI)	*P Value*
**Cardiorespiratory Fitness**							
VO_2__peak_ (l.min)	Post	0.3 (0.5)	*P* < 0.001*	0.2 (0.4)	*P* < 0.001*	0.1 (−0.1, 0.2)	*P* = *0.304*
	Follow Up	0.2 (0.5)	*P* = 0.007*	0.2 (0.5)	*P* = 0.047*	0.1 (−0.1, 0.2)	*P* = *0.573*
VO_2__peak_ (ml.kg^–1^.min^–1^)	Post	3.9 (6.0)	*P* < 0.001*	2.7 (4.4)	*P* < 0.001*	1.2 (−0.4, 2.7)	*P* = *0.130*
	Follow Up	2.9 (5.1)	*P* < 0.001*	2.0 (5.7)	*P* = 0.015*	1.0 (−0.9, 2.8)	*P* = *0.298*
Wattmax (W)	Post	12 (20)	*P* < 0.001*	7 (19)	*P* = 0.035*	6 (−1, 12)	*P* = *0.108*
	Follow Up	14 (25)	*P* < 0.001*	9 (31)	*P* = 0.023*	6 (−3, 14)	*P* = *0.178*
**Body Composition**							
Reduction in Body Mass (%)	Post	−2 (2)	*P* < 0.001*	−1 (2)	*P* = 0.008*	1 (−2,0)	*P* = *0.093*
	Follow Up	−2 (4)	*P* < 0.001*	0 (3)	*P* = *1.000*	2 (1,3)	*P* < *0.001*
BMI (kg.m^2^)	Post	−0.6 (1.0)	*P* < 0.001*	−0.3 (0.7)	*P* = 0.013*	0.3 (0.0, 0.5)	*P* = 0.050*
	Follow Up	−0.7 (1.3)	*P* < 0.001*	−0.1 (0.9)	*P* = *0.978*	0.5 (0.2, 0.8)	*P* = 0.001*
Muscle Mass (kg)	Post	−1.9 (7.3)	*P* = 0.011*	−0.7 (2.1)	*P* = *0.712*	1.2 (−0.2, 2.6)	*P* = *0.096*
	Follow Up	−1.0 (4.7)	*P* = *0.459*	−0.5 (3.0)	*P* = *1.000*	0.6 (−1.1, 2.2)	*P* = *0.490*
Fat Mass (kg)	Post	−1.5 (6.3)	*P* = 0.010*	−0.2 (2.0)	*P* = *1.000*	1.4 (0.1, 2.6)	*P* = 0.030*
	Follow Up	−0.6 (5.1)	*P* = *0.684*	0.0 (2.2)	*P* = *1.000*	0.5 (−1.0, 1.9)	*P* = *0.516*
VAT Mass (g)	Post	−30.3 (204.4)	*P* = *0.158*	19.7 (90.5)	*P* = *0.654*	50.1 (9.9, 90.2)	*P* = 0.015*
	Follow Up	−26.5 (139.6)	*P* = *0.340*	29.5 (108.5)	*P* = *0.342*	56.0 (9.4, 102.7)	*P* = 0.019*
Body Fat (%)	Post	1 (11)	*P* = *1.000*	0.1 (2)	*P* = *1.000*	1 (−1, 2)	*P* = *0.637*
	Follow Up	0.2 (5)	*P* = *1.000*	0.1 (2)	*P* = *1.000*	0.2 (−2, 2)	*P* = *0.846*
**Blood pressure**							
Systolic Blood Pressure (mmHg)	Post	−2 (10)	*P* = *0.570*	−1 (9)	*P* = *1.000*	1 (−2, 4)	*P* = *0.402*
	Follow Up	−6 (9)	*P* < 0.001*	1 (10)	*P* = *1.000*	6 (3, 9)	*P* < 0.001*
Diastolic Blood Pressure (mmHg)	Post	−1 (7)	*P* = *1.000*	2 (5)	*P* = *0.351*	2 (0, 5)	*P* = 0.050*
	Follow Up	−3 (13)	*P* = 0.049*	2 (5)	*P* = *0.127*	5 (3, 8)	*P* < 0.001*
Mean Arterial Pressure (mmHg)	Post	−1 (7)	*P* = *1.000*	1 (5)	*P* = *1.000*	2 (0, 4)	*P* = *0.064*
	Follow Up	−4 (10)	*P* = 0.001*	2 (6)	*P* = *0.216*	6 (3, 8)	*P* < 0.001*
**Glucose Tolerance**							
Fasting Glucose (mmol.L^–1^)	Post	0.0 (0.9)	*P* = *1.000*	0.1 (1.0)	*P* = *1.000*	0.1 (−0.2, 0.3)	*P* = *0.628*
	Follow Up	0.0 (0.8)	*P* = *1.000*	−0.1 (0.8)	*P* = *1.000*	0.0 (−0.3, 0.3)	*P* = *0.963*
Glucose at 60 min (mmol.L^–1^)	Post	−0.1 (2.4)	*P* = *1.000*	−0.2 (2.4)	*P* = *1.000*	0.1 (−0.7,0.8)	*P* = *0.856*
	Follow Up	−0.3 (2.5)	*P* = *1.000*	0.7 (2.4)	*P* = *0.144*	1.0 (−0.1,1.8)	*P* = 0.029*
Glucose at 120 min (mmol.L^–1^)	Post	−0.2 (2.4)	*P* = *1.000*	0.1 (1.5)	*P* = *1.000*	0.2 (−0.4, 0.8)	*P* = *0.431*
	Follow Up	−0.2 (1.9)	*P* = *1.000*	0.6 (1.8)	*P* = *0.096*	0.8 (0.1, 1.5)	*P* = 0.026*
Glucose AUC (mmol.L^–1^.120 min^–1^)	Post	1 (225)	*P* = *1.000*	10 (210)	*P* = *1.000*	6 (−56,67)	*P* = *0.854*
	Follow Up	−19 (244)	*P* = *1.000*	75 (188)	*P* = *0.081*	88 (14,162)	*P* = 0.020*
**Blood Lipids**							
Triglycerides (mmol.L^–1^)	Post	−0.1 (0.4)	*P* = *1.000*	−0.1 (0.8)	*P* = *0.423*	0.0 (−0.17, 0.18)	*P* = *0.962*
	Follow Up	−0.1 (0.5)	*P* = *0.303*	−0.1 (1.0)	*P* = *1.000*	0.1 (−0.1, 0.4)	*P* = *0.177*
Cholesterol (mmol.L^–1^)	Post	0.1 (0.7)	*P* = *0.941*	0.0 (0.7)	*P* = *1.000*	0.1 (−0.1, 0.3)	*P* = *0.339*
	Follow Up	−0.1 (0.9)	*P* = *0.942*	−0.1 (0.9)	*P* = *1.000*	0.0 (−0.3, 0.3)	*P* = *0.909*
HDL-Cholesterol (mmol.L^–1^)	Post	0.0 (0.3)	*P* = *1.000*	0.0 (0.2)	*P* = *1.000*	0.0 (0.0, 0.1)	*P* = *0.289*
	Follow Up	0.0 (0.2)	*P* = *1.000*	0.1 (0.2)	*P* = *0.506*	0.1 (0, 0.1)	*P* = *0.211*
LDL-Cholesterol (mmol.L^–1^)	Post	0.1 (0.7)	*P* = *1.000*	0.1 (0.6)	*P* = *1.000*	0.0 (−0.2, 0.2)	*P* = *0.857*
	Follow Up	0.0 (0.6)	*P* = *1.000*	−0.1 (0.7)	*P* = *1.000*	0.0 (−0.2, 0.3)	*P* = *0.737*

*VAT, visceral adipose tissue. Change compared to baseline as Mean ± SD, unless otherwise indicated, and *p* value are presented.*

### Anthropometrics

Reduction in body mass (%) (MICT *P* < 0.001, Home-HIIT *P* = 0.008) and BMI (MICT *P* < 0.001, Home-HIIT *P* = 0.013) reduced post-intervention however, only MICT maintained these reductions at follow-up (Table 4). Between group differences in reduction in body mass (%) and BMI were observed post-intervention (*P* = 0.05), with MICT reducing them more than Home-HIIT (*P* = 0.05). At follow-up a between-group difference was observed in BMI (*P* = 0.001) and body mass (*P* < 0.001), with a greater reduction seen following MICT.

Post-intervention fat mass (*P* = 0.010) and muscle mass (*P* = 0.011) were reduced in MICT, but neither reductions were maintained at follow up (Table 4). No improvements were observed in Home-HIIT at any time-point. Between-group differences in fat mass and muscle mass were observed post-intervention (*P* = 0.030), with a greater reduction seen following MICT.

### Blood Pressure

At follow-up, participants in MICT had significantly reduced systolic (*P* < 0.001), diastolic (*P* = 0.049) and mean arterial pressure (*P* = 0.001), no improvements were observed in Home-HIIT at any time-point. A between-group difference was observed at post-intervention, MICT reduced diastolic blood pressure more than Home-HIIT (*P* = 0.05). Between-group differences were also observed at follow-up for all blood pressure measurements, MICT reduced all measures compared to Home-HIIT (Table 4).

### Glucose Tolerance and Bloop Lipids

Fasting glucose, glucose concentrations during the OGTT (60- and 120 min) and glucose AUC were not improved at any time point in either group. No between-group differences were seen at post-intervention at fasting, 60- or 120 min. However, at follow-up there were significant between-group differences for glucose concentrations at 60- and 120 min, with MICT having lower glucose values compared to Home-HIIT (60 min *P* = 0.029, 120 min *P* = 0.026). There were no significant between- or within-group differences in any blood lipid markers throughout the study (Table 4).

### Intervention Characteristics, Adherence and Compliance

**FIGURE 2:**
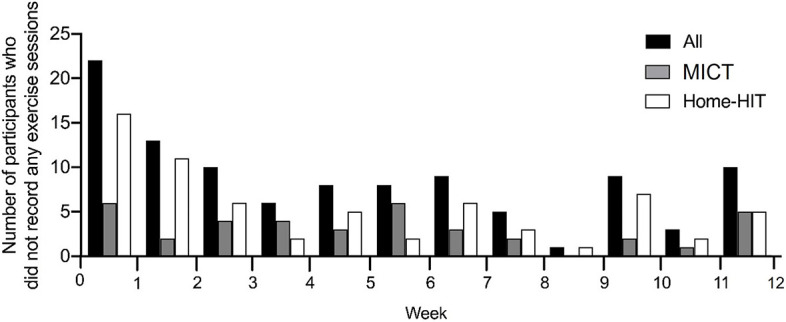
Training drop-off over the 12-week intervention period.

The mean training session duration and heart rate responses can be found in Table 5. No exercise uptake (completed 0 training sessions) was greater in Home-HIIT (*n* = 27) vs. MICT (*n* = 8). Following week 1, drop off was similar (Home-HIIT *n* = 39 vs. T-MICT *n* = 30). As a result, total drop off over the intervention period was greater in Home-HIIT (*n* = 66) than MICT (*n* = 32, Figure 2), leaving the final number of participants training at the end of the 12 week intervention as Home-HIIT (*n* = 21) and MICT (*n* = 29).

**TABLE 5:** Mean training session duration and heart rate responses during 3-month intervention of MICT or Home-HIIT.

Variable	MICT	Home-HIIT
**Duration (min:sec)**		
Intention to Treat	24:27 ± 22:03	4:14 ± 5:10
Per Protocol	50:36 ± 20:25	10:10 ± 2:00
**HR_mean_ (% HR_max_)**		
Intention to Treat	55 ± 13	−
Per Protocol	73 ± 8	−
**HR_peak_ (% HR_max_)**		
Intention to Treat	−	58 ± 20
Per Protocol	−	91 ± 7
**Time above ≥ 80% HR_max_ (min:sec)**		
Intention to Treat	−	2:40 ± 5:42
Per Protocol	−	7:45 ± 16:17
**% of Intervals Above 80% HR_max_**		
Intention to Treat	−	22 ± 31
Per Protocol	−	75 ± 25

*Intention to Treat: all training sessions were included. Per Protocol: only recorded sessions were included.*

During the 3-month intervention period adherence and compliance were similar for MICT and Home-HIIT, when using intention-to-treat or per-protocol analysis (*P* > 0.05, Table 6).

**TABLE 6:** Adherence and compliance to the 12-week MICT and Home-HIIT interventions.

	MICT	Home-HIT	*P* Value
**Intention to Treat**			
Sessions per week (n)	2 ± 1	1 ± 1	*P* = 0.114
Weekly Adherence (%)	48 ± 35	39 ± 36	*P* = 0.772
Compliance (%)	47 ± 40	30 ± 34	*P* = 0.331
**Per Protocol**			
Sessions per week (n)	2 ± 1	1 ± 1	*P* = 0.352
Weekly Adherence (%)	54 ± 32	41 ± 35	*P* = 0.528
Compliance (%)	88 ± 23	82 ± 23	*P* = 0.104

*Values presented as mean ± SD. *Intention-to-treat* analysis for all variables included all consented participants. *Per-protocol* analysis for adherence variables excluded initial dropouts (i.e., had an adherence of 0%). *Per-protocol* analysis for compliance included only recorded sessions. *Weekly adherence*: mean number of sessions per week, using 3 sessions as the maximum (i.e., 3 or 4 sessions per week would both = 100% adherence).*

### Participant Perceptions of the Exercise Interventions

Of the total 79 responses (MICT *n* = 34, Home-HIIT *n* = 45) the main barrier to previous exercise participation was motivation (*n* = 23). Home-HIIT participants frequently outlined lack of time and work/family life as barriers to exercise participation (Table 7), whereas MICT participants outlined a lack of motivation or ill-health.

**TABLE 7:** Perceived barriers to previous exercise participation, motivation behind participation and motivation for intervention choice.

	MICT	Home-HIIT
Perceived barriers to previous exercise participation	Motivation (11), Physical health (7), Time (4), Work/Family life (4), Cost/accessibility (4), Mental health (2), Perceived exercise as boring (1)	Time (12), Motivation (9), Work/family life (7), Physical health (4), Confidence (3), Knowledge (2), Cost/accessibility (2), Mental health (1), Perceived exercise as boring (1)
	*“I’m just not motivated to exercise”*	*“Time, two young children and then a busy work life meant the ‘luxury’ of going for a run/the gym was not very time productive”*
Motivation to participate	Improve health (15), Improve fitness (11), Monitor health (9), For motivation (3), Interested in research (4)	Improve health (22), Improve fitness (16), Monitor health (13), For motivation (9), Interested in research (7)
	*“The opportunity to make a positive change in relation to my health and lifestyle”*	*“I wanted to improve my muscle strength, fitness and overall health”*
Motivation for intervention choice	Already a gym member but not using the membership (9), Alternative environment (6), Use of the equipment (3), Social (2), Advice from staff (1)	Convenience (13) To save time (12), Freedom/flexibility (4), Home-based (4), No travel (3), HIIT (2), Social (1)
	*“I felt I would like to use gym equipment and have access to trained staff for help and advice.”*	*“(I have) previously been a gym member but struggled with consistency of going. Though HIIT at home would be more convenient and easier to stick to”*

*The number presented in bracket indicated the number of participants who mention this.*

Reasons for choosing Home-HIIT centered around convenience or the time-saving nature of the exercise. MICT participants stated they were gym members but not using the membership and/or that they needed an alternative environment than their home for exercise as key reasons for their choice.

Based on the survey responses, three key themes, and further subthemes were developed: (1) Health, with two subthemes, (i) motivation to start the exercise program and (ii) health outcomes of the program (2) Convenience with two subthemes, (i) motivation for exercise choice and (ii) adherence throughout the program and (3) Motivation, with three subthemes, (i) social support during the program, (ii) personalized health and exercise monitoring and (iii) achievement or satisfaction during/following the program (Table 8).

**TABLE 8:** Summary of participant responses in qualitative survey.

Theme	Subtheme	MICT		Home-HIIT	
		Positive Responses	Negative Responses	Positive Responses	Negative Responses
Health (173)	Motivation to start the program (89)	Improve health/fitness (28), Monitor Health/Fitness (6)		Improve Health/Fitness (42), Monitor Health/Fitness (13)	
		*“Lose weight, improve my fitness, and overall health in the long term”*		*“I wanted to gain a greater in depth knowledge of my own health and hope exercise can help improve it”*	
	Health outcomes of the program (84)	Physical health benefits (25), Mental health benefits (5)	Illness or Injury – Missed sessions (4), Needed to adapt exercises because of injury or illness (3)	Physical health benefits (15), Mental health benefits (5)	Illness or Injury – Missed sessions (18), Need to adapt exercises because of injury or illness (9)
		*“Made me feel more energetic, younger, less baggage to carry and feel better overall with loads more energy”*	*“My swimming pool has been closed for 6 weeks which has affected my ability to exercise when my knee won’t let me do load bearing exercises.”*	*“I noticed that I was improving my overall stamina and recovering from exercise more quickly.”*	*“I was injured near the last few weeks, which was annoying”*

Convenience (93)	Motivation for intervention choice (38)	Already a member of a gym (13)		To fit around busy work/family commitments (25)	
		*“I chose the gym as had membership wasn’t using it”*		*“I work shifts so choose the exercise at home program to fit them in around work”*	
	Adherence throughout the program (55)	Structured program (6)	Missed sessions due to lack of time (10), Waiting for other people on the machines (2).	Quick to complete (11), Home setting (6), Ability to chose exercises (5), Flexibility of time (5)	Lack of time (6), Tired (2), Distractions in the house (2)
		*“I liked the structured approach, doing 30 min 3 times a week and having a program to follow”*	*“It is harder to continue the gym when I work more. Finishing an 8 h shift would make me want to just go home and not go the gym.”*	*“I could tailor the exercises to suit which part of the body I wanted to work on. I enjoyed the versatility and being able to work at my own pace.”*	*“Sometimes it was difficult to find time to fit 20 min of exercise into a daily routine.”*

motivation (243)	Social Support during the program (69)	Friends/Family (8), Gym buddy (7), ERS team(5)	No Social Support (11)	Family or Friends (19), Others on the program (8), Exercise Buddy (2), Researcher (2)	No Social Support (10)
		*“Training with a colleague massively helped. More with just getting me to the gym than the actual exercise”*		*“My wife, we exercise together; my daughter - watches me and encourages me and occasionally gets involved”*	
	Personalized health and exercise monitoring (59)	Post testing (12), Live feedback (10), Monitored remotely by researcher (1), Session tracking (1)	Connection issues with HR monitor (3), Fell off during swimming (2), Forgot to take HR monitor sometimes (1),	Live feedback (14), Monitored remotely by researcher (4), Session tracking (3), Post testing (2)	Connection issues with HR monitor (6)
		*“The assessments (motivated me), as this helped me to measure changes”*	*“(Whilst swimming) as you push off from the wall the rush of water can move the HR monitor. So I often had to stop to tighten it”*	*“(The monitor) helps push you because you want to see your heart rate going up into Red zones”*	“*The heart monitor didn’t always work well and it took quite a while to connect”*

	Achievement or satisfaction during and following the program (115)	Health improvements (15), Felt positive during a session (3), Completing a session (3), Reaching target HR (3)	Lack of time (15), Tiredness (4), Not completing 3 sessions per week (4), Slow health improvements (2)	Completing a session (17), Health improvements (14), Reaching target HR (8), Completing target number of sessions (2), increasing intervals (2)	Not completing 3 sessions per week (5), Lack of time (5), Tiredness (5), Lack of motivation (4), Struggled to reach target HR (3), Not able to do the exercises (1)
		*“Running for a train and realizing that I no longer get out of breath. That motivated me to continue”*	*“Sometimes struggle for time to exercise which has frustrated me”*	*“I felt positive and more energetic at the end of each exercise as it made me feel good that I achieved something (when usually sat down or have been eating junk) knowing the outcomes of exercising for my health”*	*“When I felt too tired or busy to undertake a session - I felt guilty”*

*The number presented in bracket indicated the number of participants who mention this.*

## Discussion

This is the first study to examine the effectiveness of incorporating a HIIT intervention within a primary care based ERS. Contrary to our hypothesis, we show similar increases in CRF following Home-HIIT and MICT performed as part of a traditional United Kingdom ERS, which was maintained in both groups 3-months after the ERS ended. MICT was more effective than Home-HIIT for improving body composition and blood pressure. Most participants were able to complete both Home-HIIT and MICT sessions as prescribed, with no difference in compliance. However, adherence to both prescriptions was poor, despite Home-HIIT reducing perceived barriers to exercise and being chosen by a large number of participants. Overall, our findings suggest that Home-HIIT is not superior to MICT when incorporated into a United Kingdom ERS. However, Home-HIIT did improve CRF to a similar extent as MICT and was a popular exercise prescription, suggesting it could be used to increase choice within ERS. Finally, adherence to ERS, regardless of exercise prescription, needs to be improved.

### Free-Living Adherence and Compliance to Moderate-Intensity Continuous Training and Home-Based High-Intensity Interval Training Within an Exercise Referral Scheme

A small number of studies have investigated HIIT in a free-living environment (Roy et al., 2018; Jung et al., 2020), and no previous studies have examined HIIT when incorporated as part of an ERS. The sparsity of real-world data has led public health experts to suggest that HIIT’s reach and adoption by sedentary individuals is likely to be very poor (Biddle and Batterham, 2015). Like the current study, Roy et al. (2018) used a patient preference design where overweight/obese individuals self-selected either a 12-month HIIT or MICT intervention, during which 42% of participants chose to complete HIIT. Similarly, we report more than half of participants referred (56%) opted to complete Home-HIIT over MICT. Interestingly, Home-HIIT had a significantly lower baseline CRF than MICT, suggesting that, in contrast to the prevailing view that the nature of HIIT would be a barrier to exercise naïve individuals, those with lower fitness were more likely to choose Home-HIIT than traditional guidelines using MICT. The survey responses suggest that Home-HIIT was attractive due to its time efficiency. The convenience of not having to travel to exercise facilities and carrying out exercise at home, at a time of their choosing were also acknowledged to be positive. Although the demand for Home-HIIT was greater than the MICT, the number of participants who did not record a training session or stopped training after week 1 was high. This is the first study to assess drop-off continually throughout a free-living HIIT or MICT intervention, and due to the non-randomized design it is difficult to assess if this high initial drop-out was due to the participants selecting HIIT or the demands of the HIIT intervention. Interestingly, if initial drop-outs were removed from the analysis, the difference in baseline group size was eliminated and drop-out during the remaining period was similar. Therefore Home-HIIT is an attractive option for at-risk individuals referred to an ERS, but reasons for the high initial drop out should be investigated further.

It has been suggested that sedentary/non-athletic populations, such as patients on an ERS, would not be able to successfully perform HIIT without supervision (Hardcastle et al., 2014). The per-protocol compliance shows that the majority of participants were able to perform Home-HIIT at the correct intensity (80% of sessions were completed as prescribed). As such, the current data supports previous findings which demonstrated that previously sedentary overweight/obese individuals were able to perform unsupervised HIIT at an adequate intensity (Roy et al., 2018). Therefore, there is accumulating evidence that sedentary individuals with increased CVD risk can successfully perform HIIT in a free-living environment.

Adherence to Home-HIT and MICT was low, 39 and 48%, respectively, and when compliant sessions only were considered this dropped further to 30 and 46%, respectively. Previous studies investigating adherence during free-living exercise interventions have also found adherence to be a challenge (Roy et al., 2018; Jung et al., 2020). Due to variability within reporting methods for adherence in studies assessing United Kingdom based ERS, it is difficult to compare with the current work. Taylor et al. (1998) showed that the mean attendance rate was approximately 45% during a 10 week gym-based ERS, however, patients were only prescribed 2 sessions per week. Taken together this research suggests adherence toward HIIT is not low, but adherence toward unsupervised free-living exercise programs and ERS is low. This theme was echoed in our survey responses where barriers such as tiredness, lack of motivation and work/life commitments were cited in both groups.

### Changes to Cardiorespiratory Fitness and Cardiometabolic Health

Despite low adherence to each intervention, sustained improvements in absolute and relative CRF were observed following Home-HIIT and MICT, with no difference between the prescriptions. The increase in CRF observed following Home-HIIT (11%) and MICT (15%) was greater than the reported coefficient of variation for repeated measurements of CRF within the literature (4%) (Phillips et al., 2017). These findings are supported by Jung et al. (2020) who also demonstrated significant and sustained (12-months) increases in CRF in overweight/obese individuals randomized to HIIT or MICT. Interestingly, these authors also report low adherence to both interventions (∼25–30% of prescribed minutes per week). Together these studies suggest prescription of free-living HIIT results in clinically meaningful increases in CRF, as previous work has suggested that a 1 ml.kg^–1^.min^–1^ increase in CRF was associated with a 10% reduction in cardiovascular mortality risk (Kavanagh et al., 2003) and a 45-day increase in longevity (Clausen et al., 2018).

Recent meta-analyses of supervised trials have reported similar, or superior, improvements in blood pressure (Costa et al., 2018) and reductions in fat mass (Viana et al., 2019) and visceral fat mass (Maillard et al., 2018) following HIIT interventions compared to MICT. However, in the current study participants reduced their systolic, diastolic and mean arterial blood pressure at 6-months, but only in the MICT group, resulting in a between group difference in these measures at follow-up. Importantly, the differences in systolic and diastolic blood pressure observed at follow-up (systolic −6 mmHg; diastolic −3mmHg) and between groups at follow-up (systolic 6 mmHg; diastolic 5mmHg) are likely to be clinically relevant as previous work suggests a reduction of 4–5 mmHg for systolic and 2–3 mmHg for diastolic blood pressure would be deemed clinically important in practice (Andrews et al., 2011). When comparing changes in body composition both interventions produced statistically significant reductions in body mass (% change) post-intervention, but the reduction was only maintained at follow-up in MICT, resulting in a between group difference. However, the reductions were less than the 5% associated with improvements in cardiometabolic risk, suggesting they would not be clinically relevant (Donnelly et al., 2009). A decrease in fat mass was also demonstrated following 3-months of MICT, though this reduction in fat mass was not maintained at 6-months. Although neither group reduced visceral fat mass post training or at follow-up there was also a between group difference at both time points favoring MICT. Given the importance of visceral fat in predicting cardiovascular and metabolic disease risk (Després et al., 2008; Tchernof and Després, 2013), this between group difference may be of clinical significance. Interestingly, the MICT group demonstrated a significant reduction in muscle mass post-intervention, though this reduction was not maintained at follow-up. It is unclear why this reduction was observed as previous work suggests MICT does not affect muscle mass and has been suggested as a potential addition to dietary weight management programs to preserve muscle mass (McCarthy and Berg, 2021). However, dietary records were not collected which makes interpretation of body composition data difficult. A potential reason for the discrepancy between published meta-analyses and the current study is the low adherence to HIIT, which resulted in very low weekly training volumes (∼10 min/week). In contrast, although adherence was also low in the MICT group, participants had a higher weekly training volume (∼50 min/week) which may have been enough to induce changes in these variables. Finally, neither Home-HIIT nor MICT resulted in significant improvements in blood lipids, fasting glucose, glucose tolerance or glucose AUC. Although a between group difference favoring MICT was observed for glucose tolerance at follow-up. Again these findings are in contrast to a recent meta-analysis of supervised trials that reported similar improvements in blood lipids (Wood et al., 2019) following HIIT and MICT, suggesting adherence to both Home-HIIT and MICT needed to be higher to induce changes in these outcomes. To improve these clinical outcomes, future studies need to consider how to bridge the gap between supervised exercise programs and exercise advice provided within ERS.

### Strength and Limitations

The research question led to the decision to allow participants to self-allocate intervention groups. Previous reports have questioned the potential attraction of HIIT for sedentary patients (Hardcastle et al., 2014; Biddle and Batterham, 2015) as such, we aimed to investigate participant preference for Home-HIIT and MICT prescriptions within an ERS. Participant motivation was also likely to be affected by preference, influencing adherence and therefore health outcomes (Wasmann et al., 2019). Our study was powered to detected between group differences in CRF (ml.kg.min), based on previous data comparing home-HIIT to MICT (Scott et al., 2019b), but we observed greater variability than estimated in the *a priori* power calculation and thus cannot rule out a type II error. Our decision to use two active trial arms rather than a no-treatment comparator group was informed by the research question, embedding Home-HIIT into an existing ERS and comparing changes in CRF to the traditional prescription of MICT. Given changes in CRF were observed despite low exercise adherence it cannot be ruled out that changes in habitual PA encouraged by simply enrolling in a lifestyle intervention contributed to the changes observed. HR was used to prescribe exercise intensity as the ACSM guidelines on prescription of HIIT use HR to define the work periods (REF). The rationale for using predicted HR_max_ was based on our aim to conduct a pragmatic study where the home-HIIT intervention was embedded within an existing ERS. Although United Kingdom ERS differ depending on location, to our knowledge none of them include a maximal exercise test where actual HR_max_ could be obtained. However, previous work has highlighted limitations of using HR to prescribe exercise intensity, especially during HIIT, including HR lag which can make it difficult to estimate physiological load (Buchheit and Laursen, 2013). The provision of HR monitors is not standard practice within ERS, and data from the survey suggested that participants found the feedback from the monitor an important source of motivation. Although the monitors could have influenced the intervention outcomes there is a need for additional information regarding adherence and compliance during prescribed exercise programs (Shore et al., 2019). Finally, the study presents data from a follow-up period, suggesting that improvements in CRF were maintained 3-months after the ERS. Including a follow-up period is important to understand the long term effects of the prescribed exercise. However, information is not available regarding adherence and compliance during this period. As such, it is not possible to define if this was a detraining or maintenance period, with this likely differing between participants. Future studies should look to quantify adherence and compliance during follow-up periods to better characterize this important phase.

## Conclusion

This study provides novel evidence that Home-HIIT was a viable option for patients on an ERS, and participants were able to complete Home-HIIT at the prescribed intensity. However, adherence to both Home-HIIT and MICT prescription was poor. Despite the poor adherence Home-HIIT and MICT led to sustainable and clinically relevant increases in CRF. Although improvements in CRF were seen, other measures of cardiometabolic health may have been affected by low adherence, with Home-HIIT being less effective than MICT at improving blood pressure and body composition, and neither intervention being able to improve blood lipids, fasting glucose or glucose tolerance. Together the data suggests that Home-HIIT is a viable option that could be included within ERS to increase patient choice. However, future studies need to address the poor adherence to ERS, regardless of exercise mode, investigating strategies to improve long term adherence and prevent drop-out.

## Data Availability Statement

The raw data supporting the conclusion of this article will be made available by the authors, without undue reservation.

## Ethics Statement

The studies involving human participants were reviewed and approved by Liverpool Central NHS Research Ethics Committee. The patients/participants provided their written informed consent to participate in this study.

## Author Contributions

KH, HJ, SS, FK, JS, and MC contributed to conception and design of the study. KH recruited participants and collected the data. KH, HJ, SS, AW, JS, and MC processed the cardiorespiratory fitness, body composition, cardiovascular, and metabolic data. KH and FK processed the qualitative survey responses. KH wrote the first draft of the manuscript. All authors contributed to manuscript revision, read, and approved the submitted version.

## Conflict of Interest

The authors declare that the research was conducted in the absence of any commercial or financial relationships that could be construed as a potential conflict of interest.

## Publisher’s Note

All claims expressed in this article are solely those of the authors and do not necessarily represent those of their affiliated organizations, or those of the publisher, the editors and the reviewers. Any product that may be evaluated in this article, or claim that may be made by its manufacturer, is not guaranteed or endorsed by the publisher.

## Footnotes

1
www.google.co.uk/forms/about/

2
https://www.polar.com/uk-en/beat

3
www.flow.polar.com

## References

[B1] Andrews R. Cooper A. Montgomery A. Norcross A. Peters T. Sharp D. (2011). Diet or diet plus physical activity versus usual care in patients with newly diagnosed type 2 diabetes: the Early ACTID randomised controlled trial. *Lancet* 378 129–139. 10.1016/s0140-6736(11)60442-x 21705068

[B2] Arsenijevic J. Groot W. (2017). Physical activity on prescription schemes (PARS): do programme characteristics influence effectiveness? Results of a systematic review and meta-analyses. *BMJ Open* 7:e012156. 10.1136/bmjopen-2016-012156 28153931 PMC5293992

[B3] Biddle S. J. Batterham A. M. (2015). High-intensity interval exercise training for public health: a big HIT or shall we HIT it on the head? *Int. J. Behav. Nutr. Phys. Act.* 12:95. 26187579 10.1186/s12966-015-0254-9 PMC4506613

[B4] Buchheit M. Laursen P. B. (2013). High-intensity interval training, solutions to the programming puzzle. *Sports Med.* 43 927–954. 10.1007/s40279-013-0066-5 23832851

[B5] Chinn D. J. White M. Harland J. Drinkwater C. Raybould S. (1999). Barriers to physical activity and socioeconomic position: implications for health promotion. *J. Epidemiol. Community Health* 53:191. 10.1136/jech.53.3.191 10396499 PMC1756843

[B6] Clausen J. S. Marott J. L. Holtermann A. Gyntelberg F. Jensen M. T. (2018). Midlife cardiorespiratory fitness and the long-term risk of mortality: 46 years of follow-up. *J. Am. Coll. Cardiol.* 72 987–995. 10.1016/j.jacc.2018.06.045 30139444

[B7] Costa E. C. Hay J. L. Kehler D. S. Boreskie K. F. Arora R. C. Umpierre D. (2018). Effects of high-intensity interval training versus moderate-intensity continuous training on blood pressure in adults with pre-to established hypertension: a systematic review and meta-analysis of randomized trials. *Sports Med.* 48 2127–2142. 10.1007/s40279-018-0944-y 29949110

[B8] Després J.-P. Lemieux I. Bergeron J. Pibarot P. Mathieu P. Larose E. (2008). Abdominal obesity and the metabolic syndrome: contribution to global cardiometabolic risk. *Arterioscler. Thromb. Vasc. Biol.* 28 1039–1049. 10.1161/atvbaha.107.159228 18356555

[B9] Donnelly J. E. Blair S. N. Jakicic J. M. Manore M. M. Rankin J. W. Smith B. K. (2009). American College of Sports Medicine Position Stand. Appropriate physical activity intervention strategies for weight loss and prevention of weight regain for adults. *Med. Sci. Sports Exerc.* 41 459–471. 10.1249/mss.0b013e3181949333 19127177

[B10] Gibala M. J. Little J. P. Macdonald M. J. Hawley J. A. (2012). Physiological adaptations to low-volume, high-intensity interval training in health and disease. *J. Physiol.* 590 1077–1084. 10.1113/jphysiol.2011.224725 22289907 PMC3381816

[B11] Hardcastle S. J. Ray H. Beale L. Hagger M. S. (2014). Why sprint interval training is inappropriate for a largely sedentary population. *Front. Psychol.* 5:1505. 10.3389/fpsyg.2014.01505 25566166 PMC4274872

[B12] Hoare E. Stavreski B. Jennings G. Kingwell B. (2017). Exploring motivation and barriers to physical activity among active and inactive Australian adults. *Sports* 5:47. 10.3390/sports5030047 PMC5968958 29910407

[B13] Jung M. Locke S. Bourne J. Beauchamp M. Lee T. Singer J. (2020). Cardiorespiratory fitness and accelerometer-determined physical activity following one year of free-living high-intensity interval training and moderate-intensity continuous training: a randomized trial. *Int. J. Behav. Nutr. Phys. Act.* 17 1–10. 10.1186/s12966-020-00933-8 32102667 PMC7045584

[B14] Kavanagh T. Mertens D. J. Hamm L. F. Beyene J. Kennedy J. Corey P. (2003). Peak oxygen intake and cardiac mortality in women referred for cardiac rehabilitation. *J. Am. Coll. Cardiol.* 42 2139–2143. 10.1016/j.jacc.2003.07.028 14680741

[B15] Lee D.-C. Artero E. G. Sui X. Blair S. N. (2010). Mortality trends in the general population: the importance of cardiorespiratory fitness. *J. Psychopharmacol.* 24 27–35. 10.1177/1359786810382057 PMC2951585 20923918

[B16] Maillard F. Pereira B. Boisseau N. (2018). Effect of high-intensity interval training on total, abdominal and visceral fat mass: a meta-analysis. *Sports Med.* 48 269–288. 29127602 10.1007/s40279-017-0807-y

[B17] McCarthy D. Berg A. (2021). Weight loss strategies and the risk of skeletal muscle mass loss. *Nutrients* 13:2473. 10.3390/nu13072473 34371981 PMC8308821

[B18] Miller F. L. O’connor D. P. Herring M. P. Sailors M. H. Jackson A. S. Dishman R. K. (2014). Exercise dose, exercise adherence, and associated health outcomes in the TIGER study. *Med. Sci. Sports Exerc.* 46 69–75. 10.1249/MSS.0b013e3182a038b9 23793231 PMC3867583

[B19] Morgan F. Battersby A. Weightman A. L. Searchfield L. Turley R. Morgan H. (2016). Adherence to exercise referral schemes by participants–what do providers and commissioners need to know? A systematic review of barriers and facilitators. *BMC Public Health* 16:227. 10.1186/s12889-016-2882-7 26944952 PMC4779205

[B20] Morgan O. (2005). Approaches to increase physical activity: reviewing the evidence for exercise-referral schemes. *Public Health* 119 361–370. 15780323 10.1016/j.puhe.2004.06.008

[B21] Murphy S. M. Edwards R. T. Williams N. Raisanen L. Moore G. Linck P. (2012). An evaluation of the effectiveness and cost effectiveness of the National Exercise Referral Scheme in Wales, UK: a randomised controlled trial of a public health policy initiative. *J. Epidemiol. Community Health* 66 745–753. 10.1136/jech-2011-200689 22577180 PMC3402741

[B22] National Institute for Clinical Excellence (2014). *Physical Activity: Exercise Referral Schemes.* London: National Institute for Clinical Excellence.

[B23] Pavey T. Taylor A. Hillsdon M. Fox K. Campbell J. Foster C. (2012). Levels and predictors of exercise referral scheme uptake and adherence: a systematic review. *J. Epidemiol. Community Health* 66 737–744. 10.1136/jech-2011-200354 22493474

[B24] Phillips B. E. Kelly B. M. Lilja M. Ponce-González J. G. Brogan R. J. Morris D. L. (2017). A practical and time-efficient high-intensity interval training program modifies cardio-metabolic risk factors in adults with risk factors for type II diabetes. *Front. Endocrinol.* 8:229. 10.3389/fendo.2017.00229 28943861 PMC5596071

[B25] Prior F. Coffey M. Robins A. Cook P. (2019). Long-term health outcomes associated with an exercise referral scheme: an observational longitudinal follow-up study. *J. Phys. Act. Health* 16 288–293. 10.1123/jpah.2018-0442 30892972

[B26] Ritchie J. Spencer L. (2002). “Qualitative data analysis for applied policy research,” in *Analyzing Qualitative Data*, eds Bryman A. Burgess R. (London: Routledge), 187–208. 10.4324/9780203413081-14

[B27] Rowley N. (2019). Exercise referral schemes in the UK. *ACSM’s Health Fit. J.* 23 6–8.

[B28] Roy M. Williams S. M. Brown R. C. Meredith-Jones K. A. Osborne H. Jospe M. (2018). High-Intensity interval training in the real world: outcomes from a 12-month intervention in overweight adults. *Med. Sci. Sports Exerc.* 50 1818–1826. 10.1249/MSS.0000000000001642 29683919

[B29] Scott S. N. Shepherd S. O. Hopkins N. Dawson E. A. Strauss J. A. Wright D. J. (2019b). Home-hit improves muscle capillarisation and eNOS/NAD (P) Hoxidase protein ratio in obese individuals with elevated cardiovascular disease risk. *J. Physiol.* 597 4203–4225. 10.1113/JP278062 31218680

[B30] Scott S. N. Shepherd S. O. Andrews R. C. Narendran P. Purewal T. S. Kinnafick F. (2019a). A multidisciplinary evaluation of a virtually supervised home-based high-intensity interval training intervention in people with type 1 diabetes. *Diabetes Care* 42 2330–2333. 10.2337/dc19-0871 31530660

[B31] Shore C. B. Hubbard G. Gorely T. Polson R. Hunter A. Galloway S. D. (2019). Insufficient reporting of factors associated with exercise referral scheme uptake, attendance, and adherence: a systematic review of reviews. *J. Phys. Act. Health* 16 667–676. 10.1123/jpah.2018-0341 31203705

[B32] Taylor A. H. Doust J. Webborn N. (1998). Randomised controlled trial to examine the effects of a GP exercise referral programme in Hailsham, East Sussex, on modifiable coronary heart disease risk factors. *J. Epidemiol. Community Health* 52 595–601. 10.1136/jech.52.9.595 10320861 PMC1756762

[B33] Taylor K. L. Weston M. Batterham A. M. (2015). Evaluating intervention fidelity: an example from a high-intensity interval training study. *PLoS One* 10:e0125166. 10.1371/journal.pone.0125166 25902066 PMC4406743

[B34] Tchernof A. Després J.-P. (2013). Pathophysiology of human visceral obesity: an update. *Physiol. Rev.* 93 359–404. 10.1152/physrev.00033.2011 23303913

[B35] Viana R. B. Naves J. P. A. Coswig V. S. De Lira C. A. B. Steele J. Fisher J. P. (2019). Is interval training the magic bullet for fat loss? A systematic review and meta-analysis comparing moderate-intensity continuous training with high-intensity interval training (HIIT). *Br. J. Sports Med.* 53 655–664. 10.1136/bjsports-2018-099928 30765340

[B36] Wasmann K. A. Wijsman P. Van Dieren S. Bemelman W. Buskens C. (2019). Partially randomised patient preference trials as an alternative design to randomised controlled trials: systematic review and meta-analyses. *BMJ Open* 9:e031151. 10.1136/bmjopen-2019-031151 31619428 PMC6797441

[B37] Wood G. Murrell A. Van Der Touw T. Smart N. (2019). HIIT is not superior to MICT in altering blood lipids: a systematic review and meta-analysis. *BMJ Open Sport Exerc. Med.* 5:e000647. 10.1136/bmjsem-2019-000647 PMC6937112 31921439

[B38] World Health Organization (2013). *Global Action Plan for the Prevention and Control of Noncommunicable Diseases 2013-2020.* Geneva: World Health Organization.

