# Supplementary material for: Home-Based HIIT and Traditional MICT Prescriptions Improve Cardiorespiratory Fitness to a Similar Extent Within an Exercise Referral Scheme for At-Risk Individuals
Source: Front Physiol. 2021 Nov 10;12:750283. doi: 10.3389/fphys.2021.750283 (PMC8631444; doi:10.3389/fphys.2021.750283)
Supplement: Supplementary file 1 [file Table_1.DOCX]

| Criteria | One or more of the following:   - Referred to Active Lifestyles Exercise Referral Scheme by a GP - High blood pressure - Angina (treated and stable) - Mental Health issues (anxiety/ stress/ depression) - Arthritis - Previous Heart attack or heart surgery (not under current investigation) - Impaired glucose tolerance - Overweight/ obese (BMI >25) - Aged 18-65 - Dyslipidaemia (total cholesterol   >5 mmol/L) | - Aged <18 or >65 - Cardiac rehab patient - Blood pressure >180/100 and/or uncontrolled or poorly controlled hypertension - Currently prescribed Beta-blockers - Cardiomyopathy - Uncontrolled tachycardia - Cardiac arrhythmia - Valvular heart disease - Aneurysms - Uncontrolled (drug resistant) epilepsy - failed to become (and stay) seizure free following trials of two seizure medications. - History of falls or dizzy spells in the last 12 months - Excessive or unexplained breathlessness on exertion - Uncontrolled or poorly controlled asthma (severe COPD) - Limitation in performing day-to-day activities, weekly nocturnal symptoms and awakening, more need for rescue medications, lung function (FEV1) < 80%, three or more asthma attacks per year - Pregnant or breast feeding or becomes pregnant during the study - End stage renal disease - Awaiting medical investigation - Severe mental health condition - Diabetes and 1 of the following   - Aged >35   - Type 2 diabetes mellitus >10 y duration   - Type 1 diabetes mellitus >15 y duration   - Hypercholesterolemia (total cholesterol >6.2 mmol/l)   - Hypertension (systolic blood pressure >140 or diastolic >90 mm Hg)   - Smoking   - Family history of coronary artery disease (CAD) in first-degree relative <60 y   - Presence of microvascular disease   - Peripheral vascular disease   - Autonomic neuropathy |
| --- | --- | --- |

**Supplementary Data Table 1. Inclusion and exclusion criteria.**
