# Supplementary material for: Home-Based HIIT and Traditional MICT Prescriptions Improve Cardiorespiratory Fitness to a Similar Extent Within an Exercise Referral Scheme for At-Risk Individuals
Source: Front Physiol. 2021 Nov 10;12:750283. doi: 10.3389/fphys.2021.750283 (PMC8631444; doi:10.3389/fphys.2021.750283)
Supplement: Supplementary file 2 [file Table_2.DOCX]

**Supplementary Data Table 2. Qualitative Survey Questions**

| 1. **What attracted you to take part in the programme?** |
| --- |
| **Prompts:**   - How did you hear about it? - Why did you want to become involved? |
| **2. Can you tell us about your experiences of the programmes?** |
| **Prompt:**   - Has the programme met your expectations? - What do you like about the programme? What don’t you like about the programme? - Can you give examples of when things went well / not so well? What happened? How did you overcome difficulties? - Could anything have been done differently? |
| **3. How has the programme impacted your life overall?** |
| **Prompts:**   - Benefits? - Why do you think these have (have not) occurred? Have these outcomes changed over time? |
| **4. Have you experienced any barriers to completing the exercise programme?** |
| **Prompts:**   - How were you able to overcome them? - Can you provide us with any examples of when you have faced barriers? Is there anything that you need to be able to overcome those barriers? |
| **5. Who helps you in your efforts to be active?** |
| **Prompts:**   - How do they support/help you? Can you provide us with some examples? |
| **6. What are your intentions regarding sport and exercise going forward?** |
| **Prompts:**   - What do you intend to do? - Who with and where? |
